# Supplementary figures and images for: The Candida albicans virulence factor candidalysin polymerizes in solution to form membrane pores and damage epithelial cells
Source: eLife. 2022 Sep 29;11:e75490. doi: 10.7554/eLife.75490 (PMC9522247; doi:10.7554/eLife.75490)

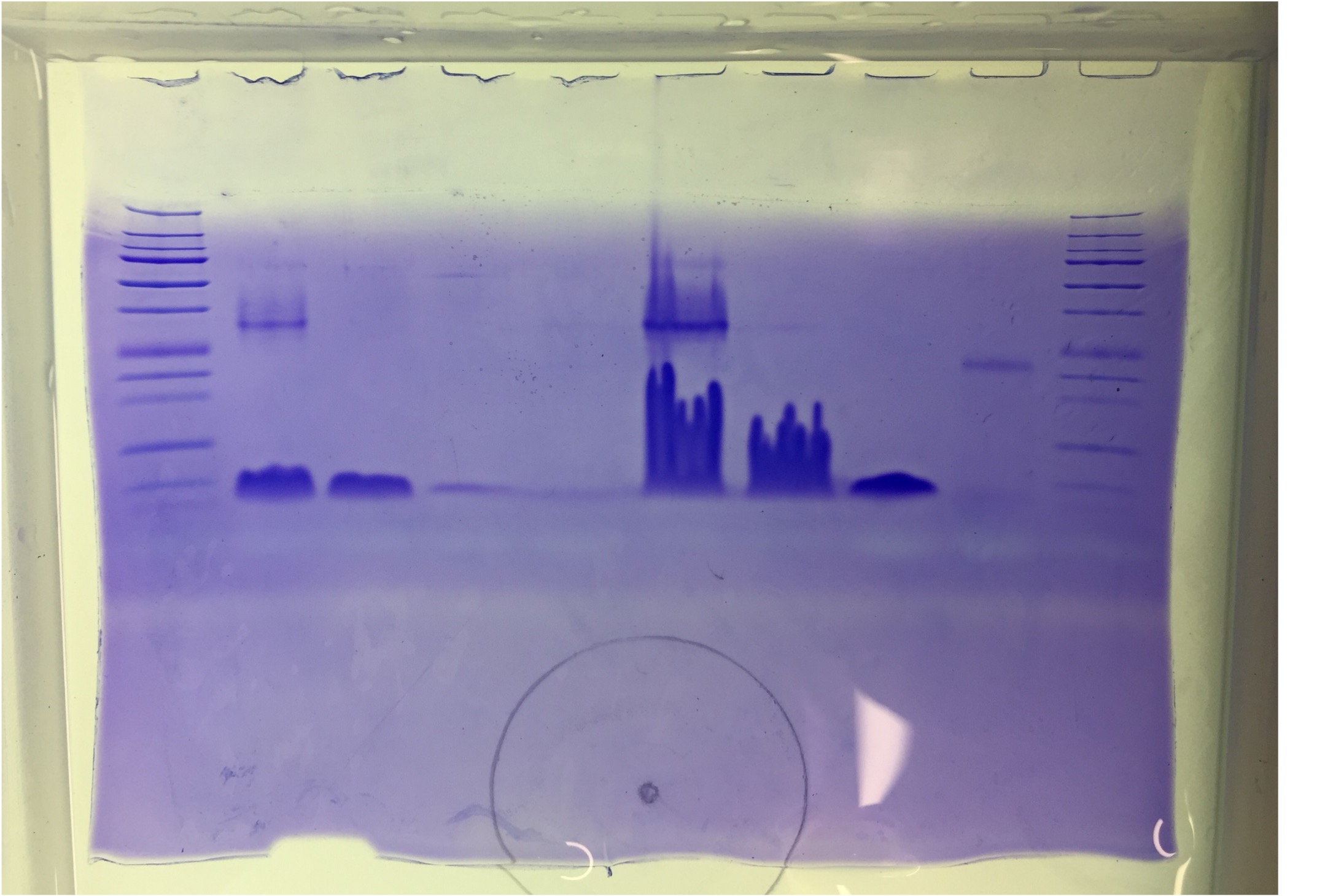

Supplement: Figure 1—source data 1. [file elife-75490-fig1-data1.zip › CL SDS PAGE.jpg]

## Slide 1
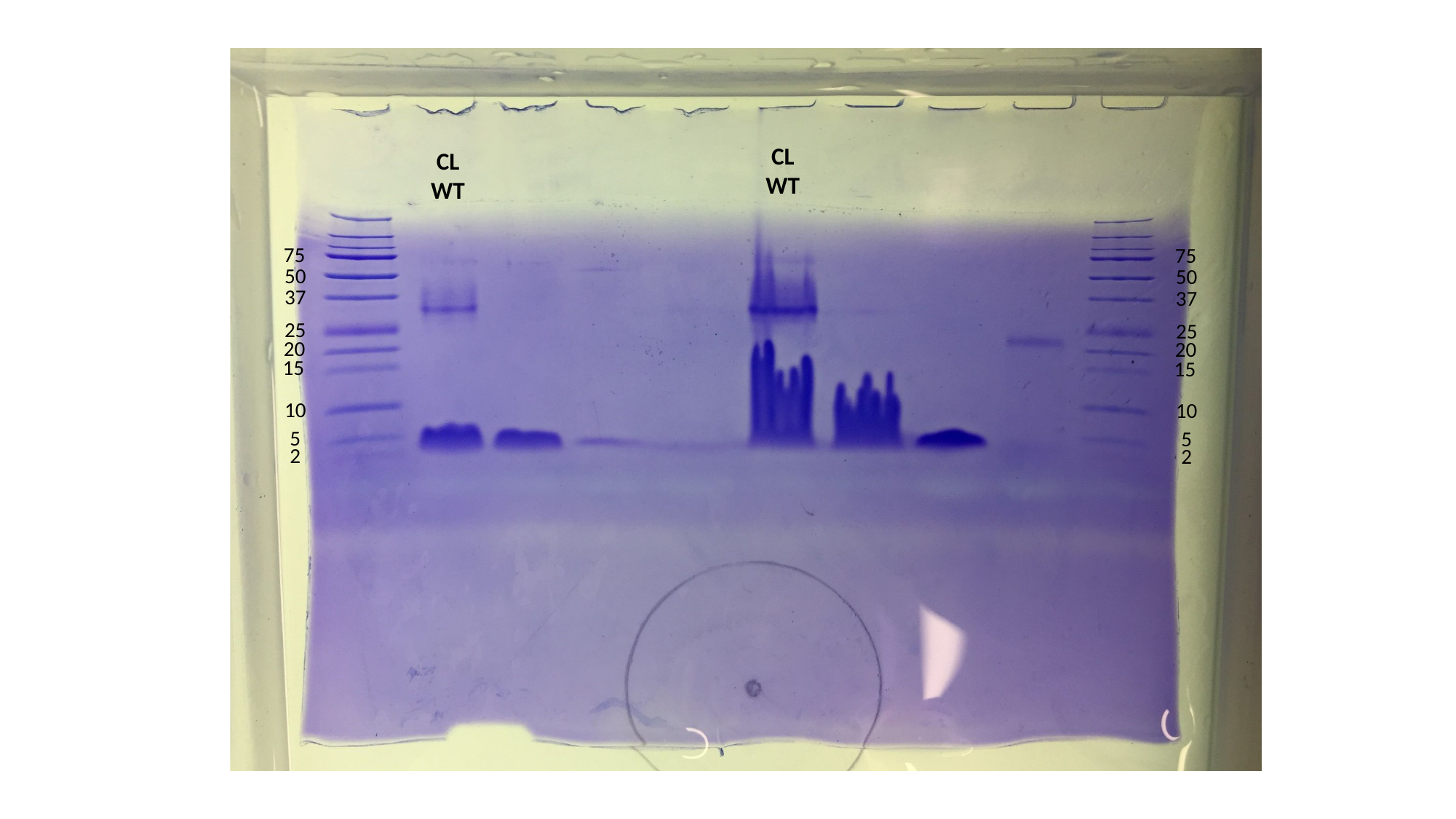

CL
WT
CL
WT
75
50
37
25
20
15
10
5
2
75
50
37
25
20
15
10
5
2

Supplement: Figure 1—source data 1. [file elife-75490-fig1-data1.zip › Figure 1_SDS PAGE_Source Data 1.pptx]

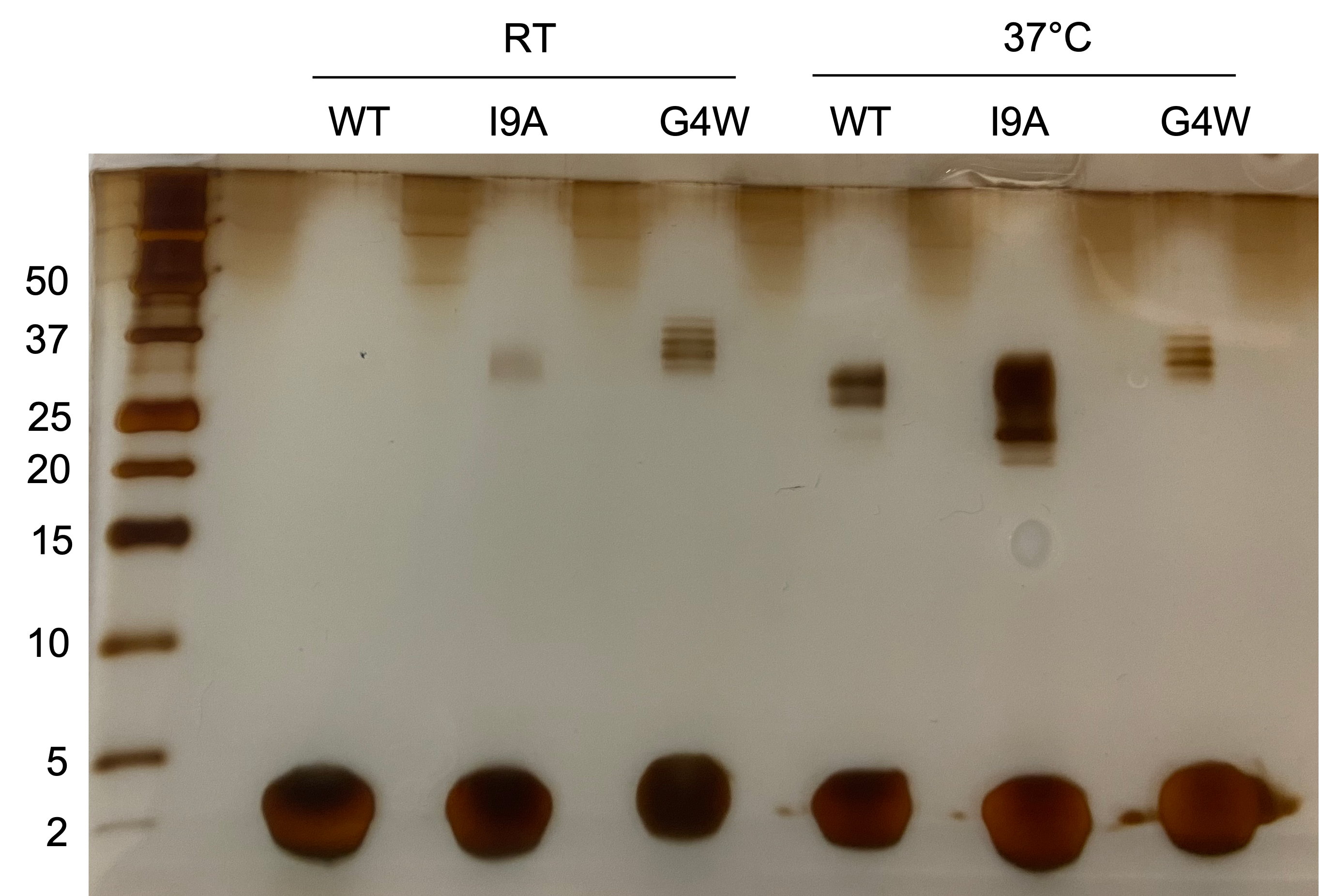

Supplement: Figure 6—source data 2. [file elife-75490-fig6-data2.zip › Figure 6_Source Data Gel.png]

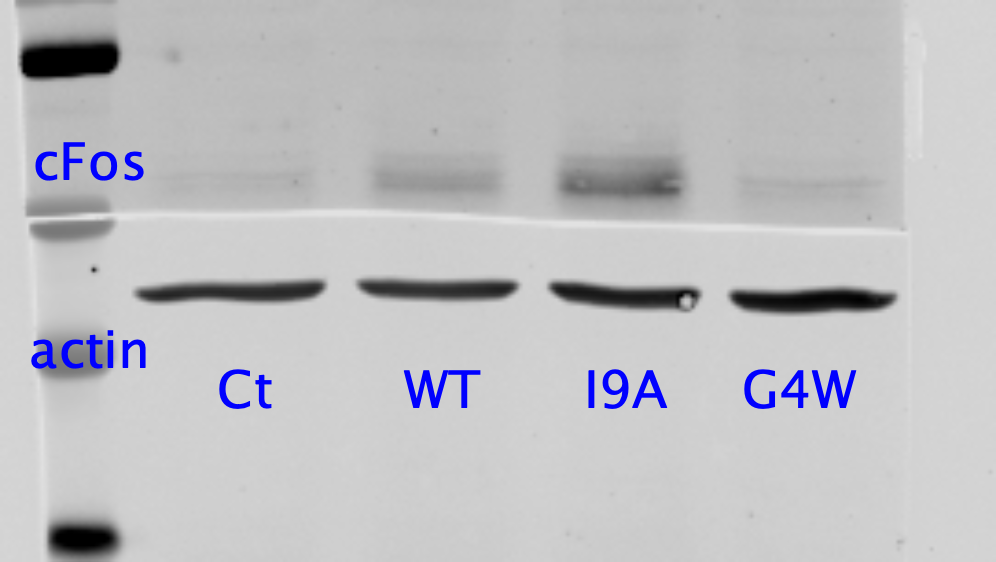

Supplement: Figure 7—source data 1. [file elife-75490-fig7-data1.zip › actin and cFos labeled WT I9A G4W Representative Image_from 1012021.png]

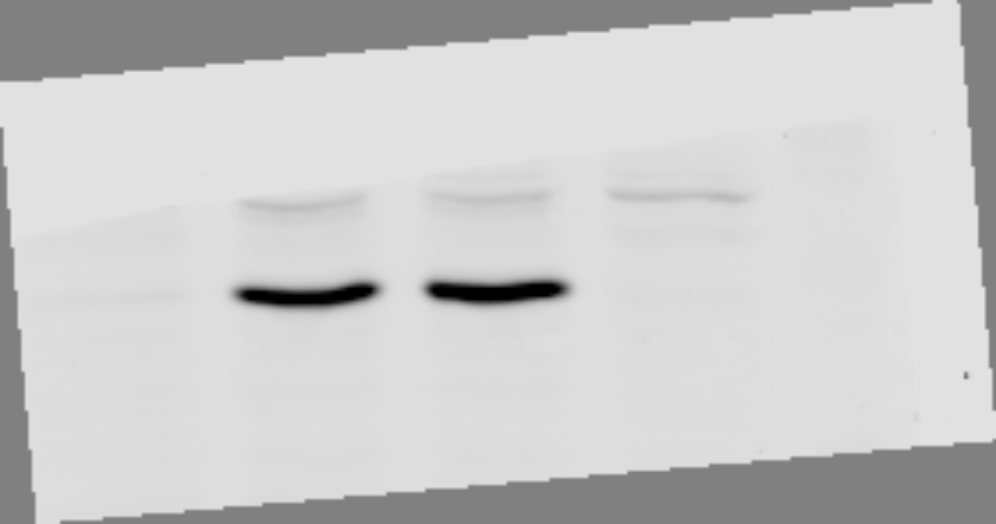

Supplement: Figure 7—source data 1. [file elife-75490-fig7-data1.zip › pMKP1 WT I9A G4W Representative Image_from 1012021.png]

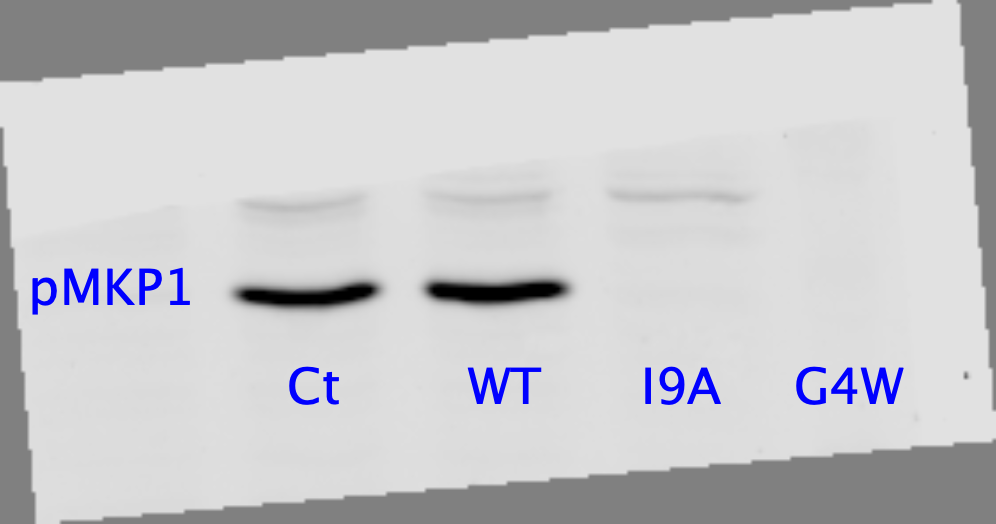

Supplement: Figure 7—source data 1. [file elife-75490-fig7-data1.zip › pMKP1 labeled WT I9A G4W Representative Image_from 1012021.png]

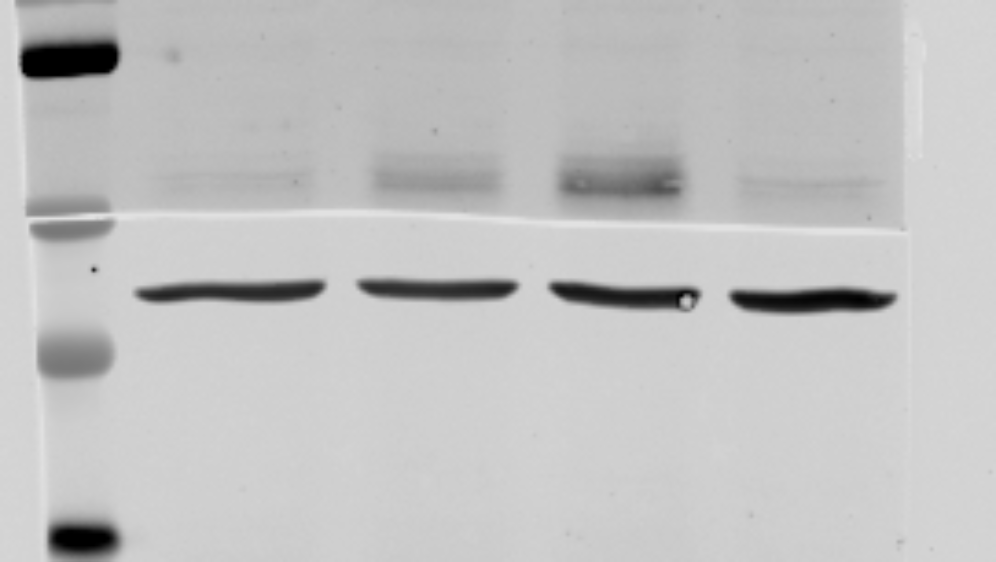

Supplement: Figure 7—source data 1. [file elife-75490-fig7-data1.zip › cFos and Actin WT I9A G4W Representative Image_from 1012021.png]
